# Age- and Sex-Dependent Variation in the Type I Interferon Signature of Healthy Individuals

**DOI:** 10.3390/medicina61122230

**Published:** 2025-12-17

**Authors:** Ilaria Galliano, Matteo Volpe, Cristina Calvi, Marzia Pavan, Anna Massobrio, Stefano Gambarino, Roberto Albiani, Claudia Linari, Anna Clemente, Anna Pau, Paola Montanari, Massimiliano Bergallo

**Affiliations:** 1 Department of Public Health and Pediatric Sciences, University of Turin, Piazza Polonia 94, 10126 Turin, Italy; matteo.volpe@edu.unito.it (M.V.); cristina.calvi@unito.it (C.C.); stefano.gambarino@unito.it (S.G.); anna.clemente@unito.it (A.C.); pauanna1@gmail.com (A.P.); paola.montanari@unito.it (P.M.); massimiliano.bergallo@unito.it (M.B.); 2 Laboratory of Specialistic Pediatry, Department of Pediatric Pathology and Care, Regina Margherita Children’s Hospital, Piazza Polonia 94, 10126 Turin, Italy; 3 Immunohematology and Transfusion Medicine, Regina Margherita Children’s Hospital, 10126 Turin, Italy; mapavan@cittadellasalute.to.it (M.P.); ralbiani@cittadellasalute.to.it (R.A.); 4 Laboratory Medicine, Regina Margherita Children’s Hospital, 10126 Turin, Italy; amassobrio@cittadellasalute.to.it (A.M.); clinari@cittadellasalute.to.it (C.L.); 5 BioMole.Srl, 10126 Turin, Italy

**Keywords:** interferon-stimulated genes (ISGs), aging, sex differences, gene expression, healthy population

## Abstract

*Background and Objectives*: Type I interferon (IFN-I) transcriptional signatures are widely utilised as readouts of innate immunity. We evaluated whether age and sex affect single interferon-stimulated genes (ISGs) and the composite IFN-I score, with implications for control selection and assay calibration. *Materials and Methods*: Ninety-five healthy individuals (53 males, 42 females; 18 days to 89 years) were studied. Whole-blood expressions of IFI27, IFI44L, IFIT1, ISG15, RSAD2 and SIGLEC1 was quantified by RT-qPCR, normalised to GAPDH and calibrated to a paediatric reference. Age associations used Spearman’s rho; sex differences, two-sided Mann–Whitney U tests. *Results*: Age effects were modest and gene-specific: IFI44L declined and IFI27 increased with age (significant overall and in females), whereas in males only IFI44L decreased; other ISGs were null (|r| ≤ 0.36). The composite IFN-I score showed no association with age or sex, indicating that aggregation mitigates small gene-level variation and that demographic influences on baseline IFN-I readouts appear minimal within this six-gene whole-blood qPCR panel in our cohort. *Conclusions*: Methodologically, a single primary cut-off within homogeneous pipelines is appropriate. Although best practice favours age-, sex- and matrix-matched healthy controls, our data show no significant age- or sex-related differences in the composite IFN-I score; matching therefore primarily supports comparability and clinical governance rather than correction of demographic shifts.

## 1. Introduction

Type I interferons (IFN-I) represent a central axis of the innate immune response, constituting the first line of defense against most viral infections and contributing to the regulation of inflammatory, autoimmune, and antitumor processes. The IFN-I family includes numerous subtypes of IFN-α, together with IFN-β, IFN-ε, IFN-κ, and IFN-ω, all characterized by their ability to bind to the heterodimeric IFNAR receptor, consisting of the IFNAR1 and IFNAR2 subunits, expressed in almost all nucleated cells [1].

Once activated, the receptor induces phosphorylation of the transcription factors STAT1 and STAT2 by the kinases JAK1 and TYK2, which together with the IRF9 factor form the ISGF3 complex. The latter translocates to the nucleus and binds to the ISRE (Interferon-Stimulated Response Element) sequences of target genes, inducing the expression of a large repertoire of genes known as Interferon-Stimulated Genes (ISGs) [2,3]. ISGs represent the molecular effector of IFN-I activity and comprise several hundred genes with antiviral, immunoregulatory, and metabolic functions, including IFI27, IFI44L, IFIT1, ISG15, MX1, OAS1, and RSAD2 [4]. Most nucleated cells are capable of producing type I interferons in response to the activation of pattern recognition receptors (PRRs) by microbial components. PRRs are distributed on the plasma membrane and within intracellular compartments of host cells, where they detect pathogen-associated molecular patterns from bacteria and viruses (TLR4, TLR2), in the cytosol (e.g., RIG-I, MDA5, and the DNA sensor cGAS with the STING adapter), and in endosomal compartments (TLR3, TLR7, TLR8). PRRs are activated by recognizing exogenous nucleic acids, self-DNA in the cytosol, and non-nucleic pathogen-associated molecular patterns (PAMPs) [5]. Type I interferons (IFN-I) belong to a class of cytokines with pleiotropic effects and perform three key functions throughout the immune response. First, they induce an antiviral/antibacterial state in target cells, limiting the spread of infectious and inflammatory agents, particularly viruses [3,6,7]. They modulate innate immunity by enhancing antigen presentation and NK cell activity, and orchestrate inflammatory pathways and cytokine production [8,9,10]. Finally, they promote adaptive immunity by promoting high-affinity responses and immunological memory formation [11].

In recent years, it has emerged that IFN-Is also participate in the etiopathogenesis of inflammatory diseases, finely regulating the relevant signaling pathways: as key regulators of specific targets, they can transmit signals that inhibit or amplify inflammation and immune-mediated responses [12,13,14,15]. In the field of infectious diseases, the activation of type 1 interferon signaling has been documented in the acute phase of pediatric viral respiratory infections (for example, in bronchiolitis caused by respiratory syncytial virus, RSV) with a consistent increase in ISGs both in the systemic compartment and in the mucous membranes of the airways, reflecting a robust innate response that is heterogeneous in terms of intensity and duration [16]. Recent evidence also indicates that, in some post-infectious contexts, the regulation of the IFN-I pathway may remain altered beyond the acute phase; Pediatric transcriptomic studies have described age-dependent variations in IFN-related gene programs in children and adolescents with post-SARS-CoV-2 sequelae, suggesting that immune development conditions both the amplitude and composition of the signature. In the field of immunology and rheumatology, the literature and clinical practice data converge in describing a pattern of hyperactivation of the IFN-I pathway that is common to several systemic diseases (e.g., SLE, Sjögren’s syndrome, systemic sclerosis, inflammatory myopathies), with a significant proportion of “IFN-high” patients. On the monogenic defect side, type I interferonopathies are innate immune errors characterized by chronic activation of the IFN-I pathway and persistently elevated ISG signatures; They fall within a few recurring pathogenetic axes, including defects in nucleic acid recognition/metabolism (Aicardi–Goutières spectrum) and gain-of-function variants associated with Singleton–Merten syndrome [17]. The interferon gene signature was initially defined in SLE and used to estimate IFN-related inflammation and stratify patients toward IFN-targeted therapies. Since then, coordinated quantification of ISGs has become standard practice in research and in the clinical–translational setting for the functional classification of conditions characterized by IFN-I dysregulation [17,18]. The type 1 interferon signature (IFN-I signature) is a transcriptional read-out of IFN-I pathway activation obtained by measuring a compact panel of ISGs in a coordinated manner—in clinical and translational practice often IFI27, IFI44L, IFIT1, ISG15, RSAD2, and SIGLEC1—and aggregating the expressions into an IFN-I score calculated relative to healthy controls after normalization on appropriate housekeeping genes [19,20]. The choice of a transcriptional measure stems from the fact that circulating IFN-Is are low in concentration and have a short half-life, while ISGs provide a more stable and sensitive indicator of cellular exposure to the IFNAR/JAK-STAT signal [21]. From a methodological point of view, inter-laboratory comparability of the score is challenging: panels, housekeeping, platforms, and control schemes may vary between centers, introducing systematic biases [19].

The literature indicates that healthy control groups are generally constructed to reflect the characteristics of the study population. In this work, we evaluated the effect of age and sex on the interferon signature (IFN-I) in healthy subjects, with the dual objective of identifying the most appropriate control group for determining the IFN signature and verifying whether a pediatric cohort can serve as a cross-sectional reference. To this end, we quantified the transcripts of the standard ISG panel (IFI27, IFI44L, IFIT1, RSAD2, SIGLEC1, ISG15) and calculated the IFN-I score, using a pool of pediatric subjects (0–10 years) as a reference. It is important to note that all conclusions in this study refer specifically to a six-gene IFN-I panel quantified by qPCR in whole blood.

The novelty of this study lies in three complementary aspects. First, although type I interferon signatures are widely used in research and clinical diagnostics, few studies have systematically assessed whether age or sex influence the expression of the standard six-ISG panel most commonly implemented in routine IFN-I workflows. Second, we show that the composite IFN-I score remains biologically stable across the entire lifespan, demonstrating that aggregation mitigates small gene-level variations and supporting the use of a single primary cut-off in homogeneous analytical pipelines. Third, we evaluate whether a pediatric cohort can function as a cross-sectional reference for assay calibration—a question that, to our knowledge, has not been directly addressed in qPCR-based whole-blood IFN-I protocols. Together, these features provide methodological insights with immediate implications for the selection of healthy controls and the harmonization of IFN-I measurement across laboratories.

## 2. Materials and Methods

### 2.1. Study Population

This study was conducted at Regina Margherita Hospital (Turin, Italy) and enrolled 95 participants (53 males, 42 females) aged 18 days to 89 years (IQR 10.32–51.42 years; mean 31.32 years). Participants were evaluated at Regina Margherita Hospital or at the City of Health and Science University Hospital of Turin during routine clinical assessments. Eligible individuals were uninfected patients with normal laboratory results. Exclusion criteria included suspected or confirmed infections, malignancies, interferonopathies, active inflammatory diseases, autoimmune diseases, neurological disorders, or abnormal laboratory findings.

All participants underwent routine laboratory evaluations, predominantly in outpatient settings (e.g., pre-surgical screening, pediatric metabolic or growth monitoring, or general health checks). Medical records were reviewed to exclude individuals with chronic inflammatory, autoimmune, oncologic, metabolic, or endocrine disorders. Patients receiving immunomodulatory, corticosteroid, or hormonal therapies were not eligible. No subject had documented viral or bacterial infections, positive COVID-19 tests, or vaccinations within the preceding 4 weeks. Minor non-inflammatory conditions (e.g., atopy without symptoms, mild controlled asthma) were recorded but not prevalent. Additional demographic and clinical characteristics are reported in Table 1.

### 2.2. Sample Storage

Within one hour from collection, samples were stored in RNA- stabilizing solution as following: 200 μL of whole blood were added to 800 μL of RNApro solution (BioMole, Turin, Italy) in a 1.5 mL tube and nasal swabs were resuspended in 1 mL of RNApro solution (BioMole, Turin, Italy) in a 1.5 mL tube [21]; samples were vortexed and stored at −80 °C until extraction.

### 2.3. RNA Extraction

Total RNA was isolated from whole blood using the Maxwell^®^ automated extractor (Promega, Madison, WI, USA) with the RNA Blood Kit, which includes on-board DNase digestion. Each extraction run incorporated a sterile-water negative control to monitor potential contamination. RNA yield and purity were evaluated by UV spectrophotometry at 260/280 nm, with concentrations calculated via the Beer–Lambert law. Measurements were performed with 1 µL of RNA on an ND-1000 NanoDrop spectrophotometer (Thermo Fisher Scientific, Waltham, MA, USA) at room temperature; A260/A280 ratios of 1.8–2.1 were taken as indicative of high purity.

### 2.4. Transcription Levels of Type I IFN Signatures by RT-PCR

The housekeeping gene GAPDH was adopted as a reference in all determinations, as it is one of the most stable normalisation genes, as demonstrated by previous studies [16,22,23]. The relative expression of IFN-I-induced ISG transcripts (IFI27, IFI44L, ISG15, IFIT1, RSAD2, and SIGLEC1) was calculated as described in detail previously [23]. In brief, 50 ng of total RNA was subjected to reverse transcription and amplification in a single step (one-step RT-qPCR) using the IFN-I mRNA expression kit (BM-023, BioMole), which allows the evaluation of the six ISGs indicated. The reactions were performed on a 96-well plate with the following thermal profile: 50 °C for 10 min, 95 °C for 10 min, then 40 cycles of 95 °C for 10 s and 60 °C for 30 s; each sample was analyzed in triplicate. The integrity and adequacy of the RNA extracts were verified by examining the cycle threshold (Ct) of GAPDH in all samples; since the Ct values fell within a range of good repeatability, the extracts were considered suitable for amplification.

Relative quantification was performed using the ΔΔCt method [24], and results were expressed as relative expression (RE) in arbitrary units. Briefly, after normalization of each target gene to the housekeeping gene, the method includes additional calibration of this value with the median expression of the same gene in the pediatric subset of our cohort (0–10 years), which served as the internal reference group required by the ΔΔCt method.

The IFN-I score was calculated as the median relative expression (RE) of the selected interferon-stimulated genes (IFI27, IFI44L, IFIT1, ISG15, RSAD2 e SIGLEC1). This score provides an overall measure of interferon pathway activation, serving as an indirect marker of the host antiviral transcriptional activity.

All analyses were performed in a laboratory of biosafety level 2 (BSL-2), according to the NIH and WHO guidelines [25,26].

### 2.5. Statistical Analysis

Statistical analyses began with a Shapiro–Wilk test of each marker and the IFN-I score to evaluate distributional normality; subsequent tests were chosen as parametric or non-parametric based on these results. Our data showed a nonparametric distribution.

The Mann–Whitney test, a nonparametric method based on ranks, was used for compare the transcriptional level of ISGs and IFN-I score between males and females groups in our sample.

Spearman’s correlation test evaluated the correlations of mRNA levels between ISGs and IFN-I score and age. Statistical analyses were performed using Prism, Version 9 (GraphPad Software), with significance set at *p* < 0.05.

## 3. Results

### 3.1. Influence of Age on IFN-I Signature Scores and on Expression Levels of Interferon-Stimulated Genes (IFI27, IFI44L, IFIT1, ISG15, RSAD2, SIGLEC1)

**Figure 1:**
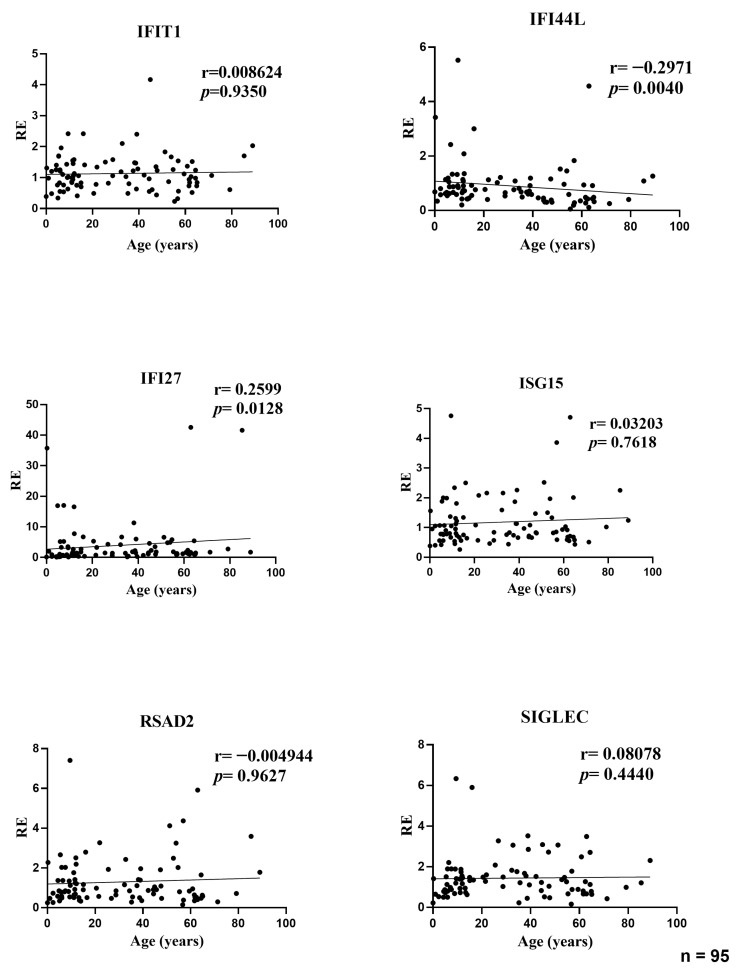
Correlations between age and relative expression (RE) of IFI27, IFI44L, IFIT1, ISG15, RSAD2, and SIGLEC1 in whole blood from all participants (*n* = 95). RE: relative expression. Circles represent individual subjects. Lines represent simple linear regressions fitted to untransformed data for visualization only. Statistical analysis: nonparametric Spearman correlation test. IFI44L decreased with age and IFI27 increased; the other ISGs showed no significant correlations.

In the full cohort, the association between age and the relative expression (RE) of six ISGs was assessed using Spearman’s rank correlation. Two transcripts demonstrated modest yet statistically significant associations. The IFI44L decreased with age (r = −0.297, *p* = 0.004), while the IFI27 increased with age (r = 0.260, *p* = 0.0128). No statistically significant correlation was identified between the expression of IFIT1 (r = 0.009, *p* = 0.935), ISG15 (r = 0.032, *p* = 0.762), RSAD2 (r = −0.005, *p* = 0.963), or SIGLEC1 (r = 0.081, *p* = 0.444) and age. The effect sizes were consistently negligible (|r| < 0.30), and the scatterplots exhibited predominantly flat trends, with increased dispersion observed for IFI27 in younger age groups, indicating the potential impact of a small number of elevated values. In consideration of the entire dataset, the investigation reveals that age accounts for a negligible proportion of the variability in baseline ISG levels (Figure 1).

To confirm that these age–ISG associations were not confounded by sex, we performed multivariable linear regression analyses including age and sex as covariates (log-transformed ISG values). As shown in Supplementary Table S1, the associations for IFI44L (negative) and IFI27 (positive) remained significant after adjustment. No other ISGs showed independent relationships with age.

### 3.2. Influence of Age on Expression Levels of Interferon-Stimulated Genes in Female Participants

**Figure 2:**
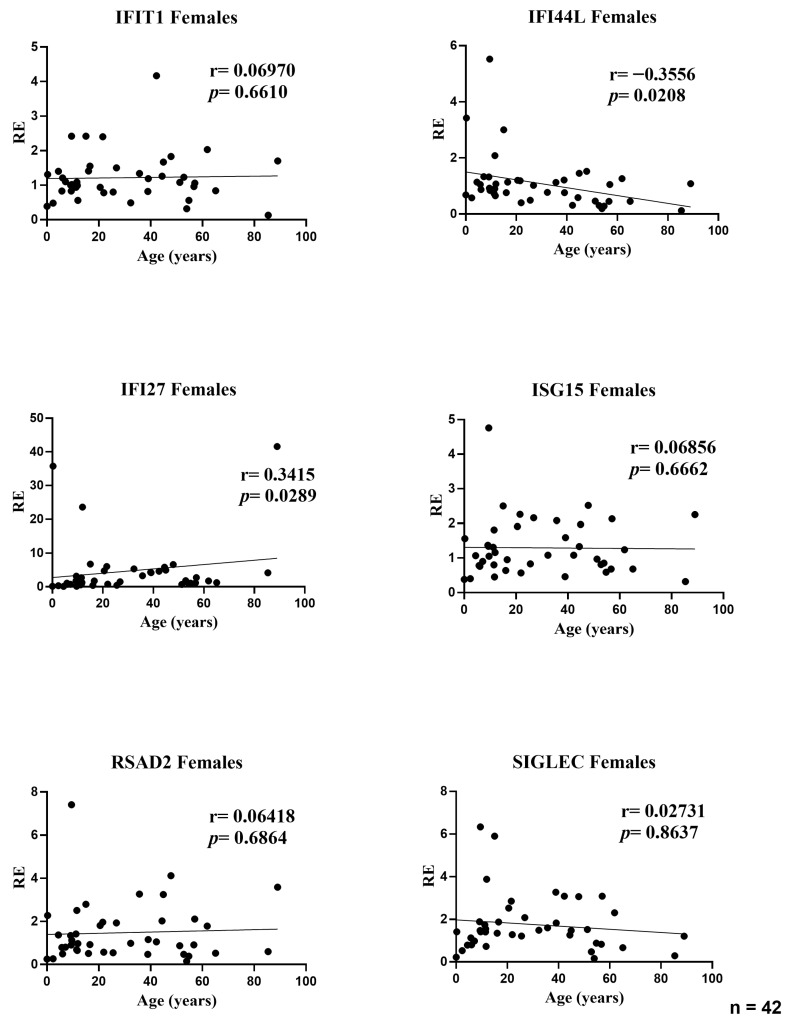
Correlations between age and relative expression (RE) of IFI27, IFI44L, IFIT1, ISG15, RSAD2, and SIGLEC1 in whole blood from female participants (*n* = 42). RE: relative expression. Circles represent individual subjects. Lines represent simple linear regressions fitted to untransformed data for visualization only. Statistical analysis: nonparametric Spearman correlation test. IFI44L decreased with age and IFI27 increased; the other ISGs showed no significant correlations.

In the female subset, age showed two modest but nominally significant associations with ISG expression: IFI44L decreased with age (r = −0.3556; *p* = 0.0208) and IFI27 increased with age (r = 0.3415; *p* = 0.0289). No age-related correlations were observed for IFIT1 (r = 0.0697; *p* = 0.6610), ISG15 (r = 0.0686; *p* = 0.6662), RSAD2 (r = 0.0642; *p* = 0.6864), or SIGLEC1 (r = 0.0273; *p* = 0.8637). Effect sizes were small (|r| ≤ 0.36), indicating that age explains only a minor fraction of between-subject variability in female ISG levels; the dispersion for IFI27 was wider at younger ages, consistent with a few high-expression values (Figure 2).

### 3.3. Influence of Age on Expression Levels of Interferon-Stimulated Genes in Male Participants

**Figure 3:**
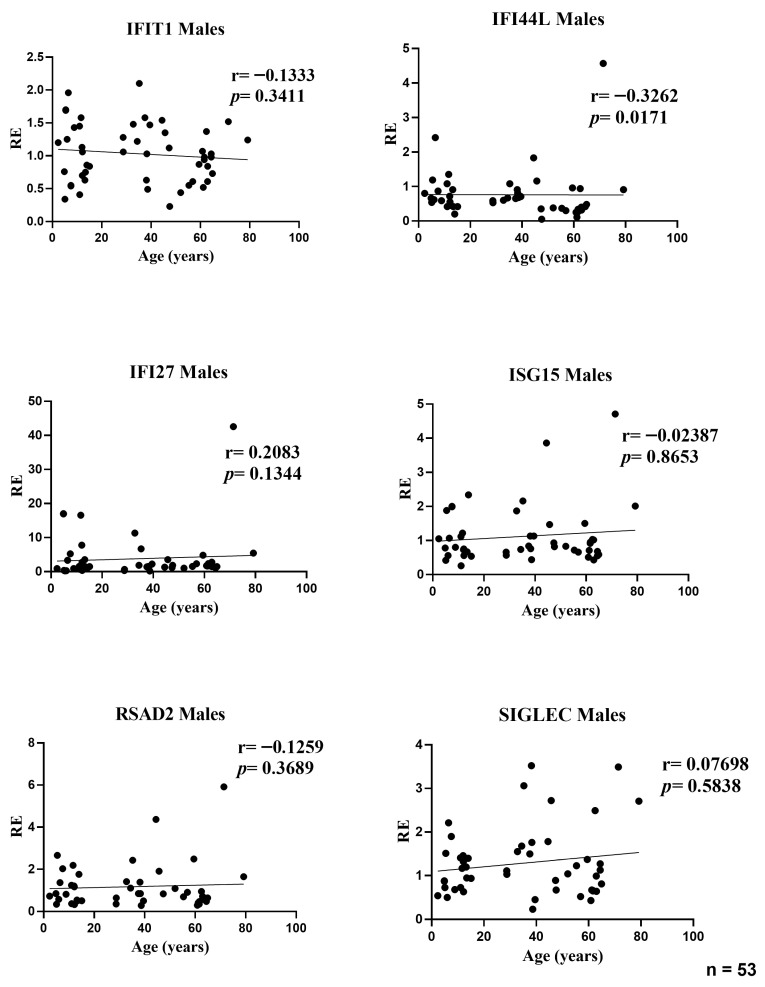
Correlations between age and relative expression (RE) of IFI27, IFI44L, IFIT1, ISG15, RSAD2, and SIGLEC1 in whole blood from male participants (*n* = 53). RE: relative expression. Circles represent individual subjects. Lines represent simple linear regressions fitted to untransformed data for visualization only. Statistical analysis: nonparametric Spearman correlation test. IFI44L decreased with age; the other ISGs showed no significant age-related correlations.

In the male subset, age was associated with a modest decline in IFI44L expression (r = −0.3262, *p* = 0.0171). No other ISGs showed significant age-related correlations: IFIT1 (r = −0.1333, *p* = 0.3411), IFI27 (r = 0.2083, *p* = 0.1344), ISG15 (r = −0.0239, *p* = 0.8653), RSAD2 (r = −0.1259, *p* = 0.3689), and SIGLEC1 (r = 0.0770, *p* = 0.5838). Effect sizes were small (|r| ≤ 0.33), indicating that age explains only a minor fraction of inter-individual variability in male ISG levels; the positive trend for IFI27 did not reach significance (Figure 3).

### 3.4. Influence of Age on the Composite IFN-I Signature Score (Overall and Sex-Stratified Analyses)

**Figure 4:**
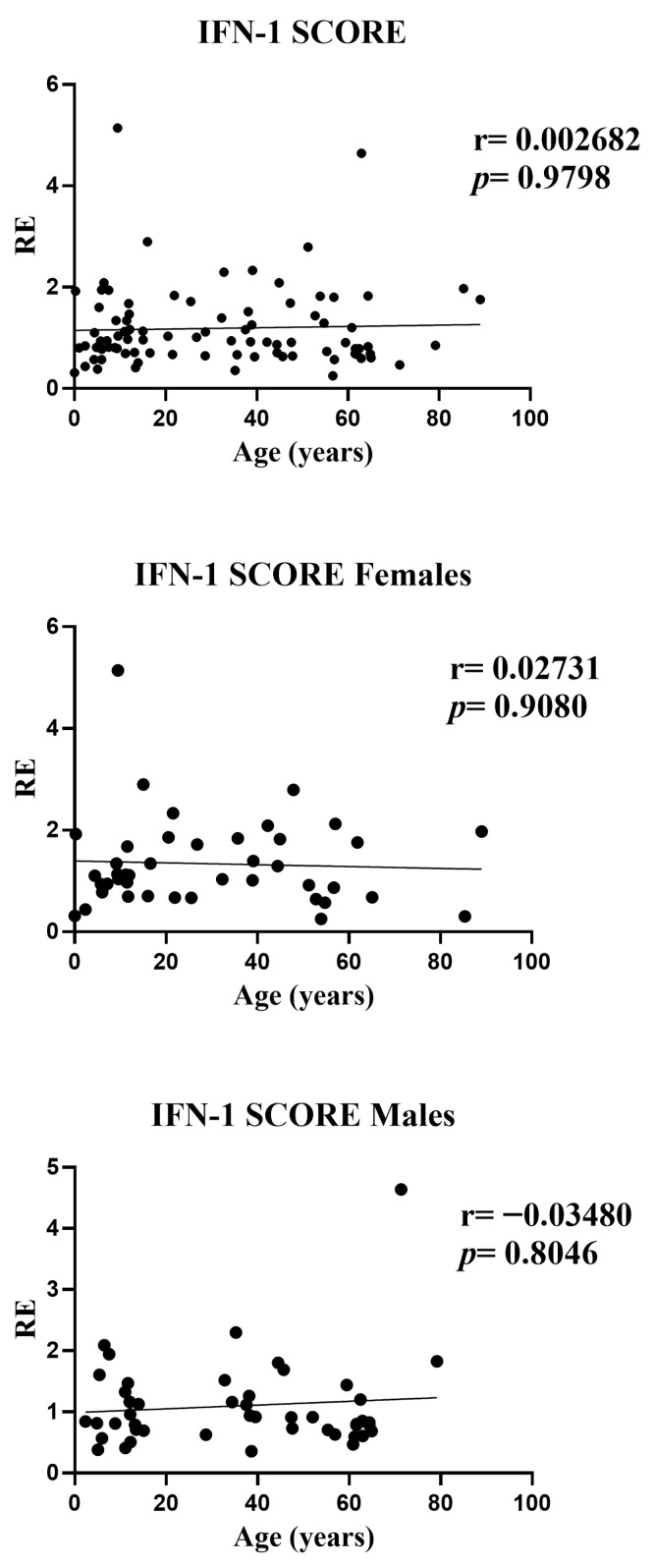
Correlations between age and the composite IFN-I score in whole blood from all participants (*n* = 95; females *n* = 42 and males *n* = 53). RE: relative expression. Circles represent individual subjects. Lines represent simple linear regressions fitted to untransformed data for visualization only. Statistical analysis: nonparametric Spearman correlation test. No significant age-related association was observed in any group.

In the overall analysis, the composite IFN-I score showed no association with age (r = 0.0027, *p* = 0.9798). Sex-stratified analyses yielded the same null pattern: females (r = 0.0273, *p* = 0.9080) and males (r = −0.0348, *p* = 0.8046). Effect sizes were close to zero and the fitted trends were essentially flat across the age range, indicating that—despite the modest gene-level signals observed for IFI27 (positive) and IFI44L (negative)—their aggregated metric does not exhibit an age gradient. Scatterplots displayed a few high values at younger ages, but these had minimal influence on the rank-based (Spearman) estimates. Overall, the IFN-I score appears largely age-independent and consistent across sexes (Figure 4).

### 3.5. Sex-Based Comparison of ISG Expression and IFN-I Score by Mann–Whitney U Test

**Figure 5:**
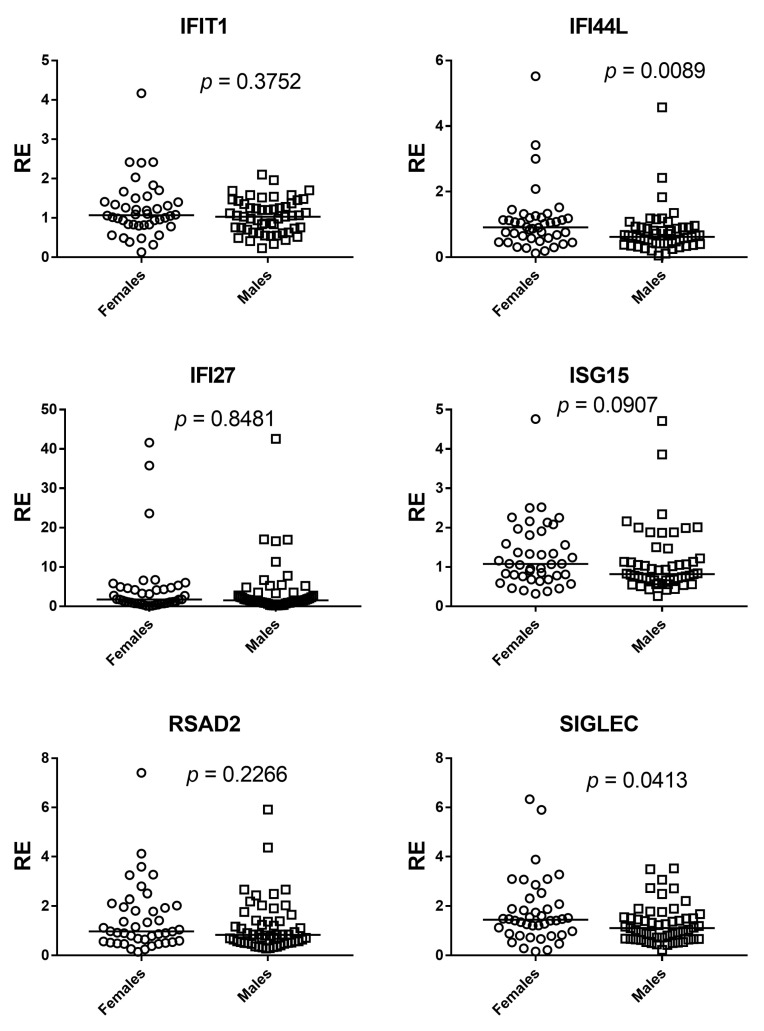
Sex comparisons of ISG expression by Mann–Whitney U test. Scatter dot plots show relative expression (RE) of IFIT1, IFI27, IFI44L, ISG15, RSAD2, and SIGLEC1 in females (circles) and males (squares). Horizontal bars indicate medians. Significant between-sex differences were observed for IFI44L (*p* = 0.0089) and SIGLEC1 (*p* = 0.0413), whereas IFIT1 (*p* = 0.3752), IFI27 (*p* = 0.8481), and RSAD2 (*p* = 0.2266) showed no differences; ISG15 displayed a non-significant trend (*p* = 0.0907).

**Figure 6:**
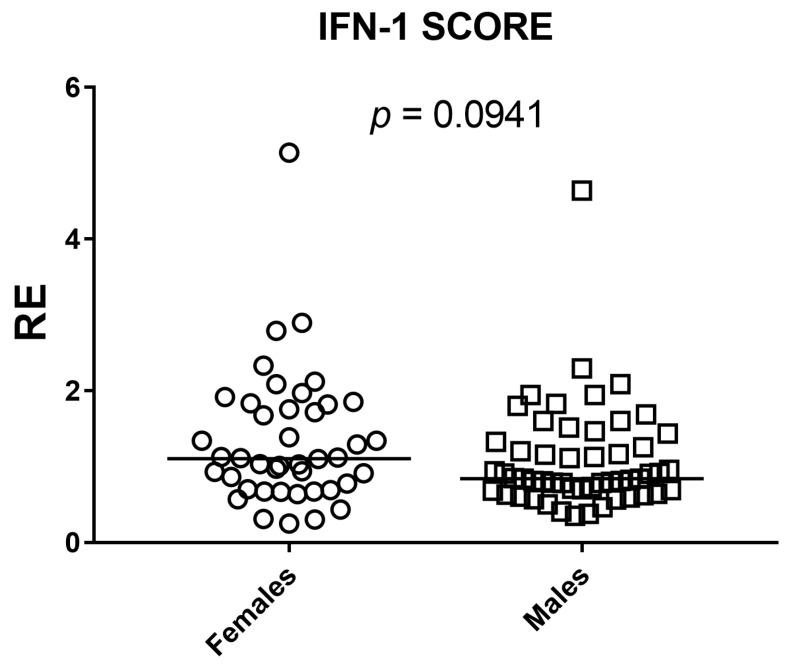
Sex comparison of the composite IFN-I score by two-sided Mann–Whitney U test. Scatter dot plot shows relative expression (RE) in females (*n* = 42) and males (*n* = 53); squares denote individual subjects and horizontal bars indicate medians. No significant between-sex difference was detected (*p* = 0.0941).

Sex differences were evaluated with two-sided Mann–Whitney U tests for each ISG and for the composite IFN-I score across 95 participants (42 females, 53 males). Gene-wise, IFI44L (*p* = 0.0089) and SIGLEC1 (*p* = 0.0413) showed statistically significant between-sex differences. For IFIT1, IFI27, and RSAD2, the estimated between-sex differences were close to zero and *p* values were well above 0.05, providing no evidence of a sex effect in these markers. ISG15 showed a weak shift in ranks suggesting a possible difference, but the evidence was insufficient (*p* = 0.0907). The composite IFN-I score mirrored the gene-level results: distributions overlapped extensively and the small median shift did not reach significance (*p* = 0.0941). Overall, any sex-related modulation appears gene-specific and, where present, of small magnitude (Figure 5 and Figure 6).

As a sensitivity analysis, we performed multivariable linear regression models in GraphPad Prism 9 using log-transformed ISG values as dependent variables and age (continuous) and sex (binary: 0 = female, 1 = male) as covariates. Parameter estimates and *p*-values are reported in Supplementary Table S1.

## 4. Discussion

Our results show that the composite type I interferon (IFN-I) score remains low across the cohort and has no systematic association with age or sex. Small gene-level shifts (an increase of IFI27 with age, a decrease of IFI44L, and modest sex differences for IFI44L and SIGLEC1) do not alter the aggregate index. We therefore conclude that, within this six-gene whole-blood qPCR panel, the composite IFN-I score appears stable across the age and sex range examined and remains robust to demographic variation, supporting the use of a single primary cut-off for within-study comparisons in a homogeneous pipeline, with clinical calibration based on age-, sex-, and matrix-matched healthy controls. A further objective of our study was to assess whether a pediatric cohort can serve as an appropriate cross-sectional reference for calibration of the IFN-I score. In our qPCR workflow, the pediatric subset of the cohort (0–10 years) was used as the internal reference group required by the ΔΔCt method. This subset included a sufficient number of individuals to provide a stable normalization factor, consistent with published evidence indicating that reference materials derived from approximately 15–20 healthy donors yield robust and reproducible ΔΔCt calibrators across analytical runs. Importantly, in our dataset pediatric values fell within the same low baseline range observed in adults, and the composite IFN-I score showed no age-related gradient. This supports the analytical suitability of pediatric samples as reference material within homogeneous pipelines, particularly for qPCR-based implementations using internal calibrators. However, while pediatric samples perform adequately for normalization, the definition of clinical cut-offs should continue to rely on larger healthy control cohorts matched for age, sex, and matrix, in line with current EULAR-aligned recommendations. Thus, pediatric reference material can be used to stabilize assay calibration, but broader pediatric datasets remain necessary for establishing universally transferable diagnostic thresholds. This interpretation is reinforced by prior evidence: Rice et al. used the canonical six-ISG qPCR whole-blood panel and reported that most healthy donors remained within the reference range with no association of the composite score with age or sex, while Kim et al. validated a 28-gene NanoString panel that includes our six markers of interest (IFI27, IFI44L, IFIT1, ISG15, RSAD2, and SIGLEC1) and found no significant differences between pediatric and adult controls in the composite score [27,28]. In our healthy cohort, age effects on ISG expression were modest and gene specific. IFI44L declined with age and IFI27 increased, reaching significance in the full cohort and among females, while in males only IFI44L showed a significant decrease; all other genes were null and effect sizes were small (|r| ≤ 0.36). By contrast, the composite IFN-I score showed no association with age in the overall sample or within either sex, indicating that aggregation buffers idiosyncratic gene-level shifts. Sex comparisons by Mann–Whitney identified differences for IFI44L and SIGLEC1, with a weak, non-significant shift for ISG15, whereas IFIT1, IFI27, and RSAD2 did not differ. The lack of a sex effect for the composite score and the near-zero age correlations support the use of a single primary cut-off for the IFN-I score within a homogeneous pipeline, with calibration against age- and sex-matched healthy controls. Isolated outliers at younger ages, particularly for IFI27, suggest rare high-expression instances but had minimal influence on rank-based estimates. Overall, within this six-gene whole-blood qPCR panel, demographic influences on baseline IFN-I readouts appear small in our cohort. While such effects may matter near single-gene decision thresholds, they do not materially affect the composite index. These results were confirmed in multivariable linear regression models adjusting for sex (Supplementary Table S1), in which only IFI44L and IFI27 retained significant age associations, while no independent demographic effects were observed for the other ISGs or for the composite IFN-I score.

The modest age-related increase in IFI27 and decrease in IFI44L (r ≈ 0.32–0.34) may reflect biological differences in ISG regulation across the lifespan. IFI27 is an early-response and highly inducible ISG [4], and its slightly higher expression in younger individuals is compatible with the heightened innate immune responsiveness typically observed in childhood [16,29]. Conversely, IFI44L belongs to an antiviral module whose inducibility may change with age, and reduced activity of this pathway has been described in transcriptional studies of peripheral blood across the lifespan [30,31]. Importantly, these gene-specific shifts remained modest in magnitude and did not influence the composite IFN-I score, underscoring that aggregated metrics buffer individual-gene variability and that, within this six-gene whole-blood qPCR panel, baseline IFN-I pathway activation appears stable across the age range examined.

A large whole-blood meta-analysis reported widespread age-associated transcriptional changes, but this pattern did not extend to our targets: IFI27 and IFI44L were not consistently age-associated in peripheral blood [30]. In healthy pediatric controls, baseline IFI44L expression shows a slight age-related decrease. The effect is statistically detectable but small and largely within the reference range, indicating modest variability across childhood [31]. In healthy whole-blood datasets that include both adult and pediatric healthy controls, IFI27 remains at baseline levels and does not exhibit a reproducible age-dependent trend; any differences across age strata are small and not systematic [32]. By contrast, studies of the airway epithelium report higher baseline expression of type I IFN–pathway genes and ISGs in pediatric than in adult airways, underscoring that age effects are compartment specific rather than universal [33]. With respect to sex, baseline differences in healthy peripheral blood appear weak for both IFI44L and SIGLEC1. In a large cohort of healthy donors, circulating soluble SIGLEC-1 tracked genetic ancestry rather than sex, indicating that population structure, rather than sex per se, accounts for most baseline variability; accordingly, sex can be treated as a minor covariate at baseline, and ancestry-aware analyses are advisable when comparing centers or populations [33]. Consistently, in a B-cell-centric study of systemic lupus erythematosus (SLE), IFI44L was upregulated with evidence of estrogen-linked regulation, yet sex-stratified analyses of the healthy control cohort showed no association between sex and IFI44L expression, indicating that any sex effect is disease-modulated rather than constitutive at baseline [34]. More broadly, females mount stronger type I IFN responses under immune activation, providing biologic plausibility for small, context-dependent sex effects [35]. Within this framework, our observation that the composite score remains stable while individual genes exhibit modest shifts is consistent with a model in which demographic influences are buffered when signals are aggregated, but can still be relevant to interpretation near decision thresholds at the single-gene level.

These considerations, as observed in this six-gene whole-blood qPCR panel, translate into concrete analytical and clinical recommendations. For within-study analyses conducted under a homogeneous pipeline, explicit stratification by age and sex is unlikely to change conclusions materially, and modeling these variables as covariates can help preserve power. However, when defining cut-offs for clinical use or transferring assays across laboratories, a conservative approach aligned with current recommendations remains warranted. HCs matched for age, sex, and matrix should be used to establish thresholds; IQC should be implemented in every run with predefined acceptance rules; and pooled reference material can stabilize normalization where appropriate. The gray zone around decision thresholds deserves explicit handling, for example, by reporting uncertainty (e.g., confidence intervals of the composite score), defining retesting criteria, and integrating laboratory results with clinical context. Assays that rely on single-gene outputs should adjust for age and sex or adopt effect-size-informed thresholds, whereas the composite score should remain the primary clinical metric within standardized workflows that include inter-laboratory comparisons. Together, these steps promote traceability, reproducibility, and comparability, attributes essential for multicenter trials and real-world implementation. Our findings also clarify a methodological gap, within the context of this six-gene whole-blood qPCR workflow, that had not been previously addressed, namely whether demographic factors or the use of a pediatric reference calibrator materially influence the IFN-I score, thereby supporting the harmonization of qPCR-based IFN-I workflows across laboratories. Several limitations temper the generalizability of our conclusions. A limitation of the present study is the absence of PBMC counts and leukocyte subset quantification (e.g., monocytes, plasmacytoid dendritic cells), which may influence absolute ISG levels in whole blood. Although ΔΔCt normalization and the composite IFN-I score buffer much of the variability related to cell abundance, analyses adjusted for cellular composition—through flow cytometry or cell-type deconvolution—would help distinguish biological variation in baseline IFN-I activation from compositional effects. Future studies integrating these measurements could provide a more refined assessment of age- and sex-related differences in IFN-I signatures. The data pertain to healthy peripheral blood and may not extend to other tissues or inflammatory states, where baseline set points and inducibility differ. Although the age range is broad, representation at the extremes of age and across genetic ancestries may affect the precision of subgroup estimates. Pre-analytical variables and batch effects were minimized through harmonized procedures, yet residual confounding cannot be excluded. Single-gene analyses entail multiple statistical tests; future work should report effect sizes with confidence intervals and, when appropriate, control the false discovery rate to contextualize small signals. From a methodological standpoint, these findings reinforce general principles for implementing IFN-I gene signatures. Standardization relies on the consistent use of age-, sex-, and matrix-matched healthy controls for cut-off definition, the inclusion of internal quality controls in every analytical run, and the judicious use of pooled reference material to stabilize normalization across sessions. These elements, together with harmonized pre-analytical handling and explicit reporting of ISG panels, housekeeping genes, and aggregation algorithms, strengthen reproducibility and support inter-laboratory comparability. These conclusions should be interpreted within the analytical framework of this six-gene IFN-I panel measured by qPCR in whole blood, and may not extend to broader transcriptomic platforms, alternative biological matrices, or different clinical context.

### Implications for Practice

Technical recommendations issued by EULAR and other consortia emphasize that clinical implementation of IFN-I signatures requires robust procedural standardization, including traceable pre-analytical workflows, validated ISG panels, appropriate housekeeping gene selection, and clearly defined aggregation algorithms. These elements provide a practical framework for laboratories aiming to harmonize six-gene IFN-I workflows and ensure reproducibility across clinical settings.

Finally, platforms and protocols differ across laboratories; explicit reporting of ISG panels, housekeeping genes, pre-analytical handling, and aggregation algorithms will facilitate external validation and meta-analytic synthesis.

## 5. Conclusions

In conclusion, within this six-gene whole-blood qPCR panel, the apparent biological stability of the composite IFN-I score across age and sex range examined complements, rather than replaces, best practices for standardization and calibration. Within this six-gene whole-blood qPCR workflow, a single primary cut-off is defensible for within-study comparisons conducted under homogeneous conditions. However, clinical implementation and inter-laboratory transferability continue to require conservative safeguards, including the use of age-, sex- and matrix-matched healthy controls for cut-off definition, IQC in every analytical run, and the judicious use of pooled reference material to stabilize normalization. Framed in this way, the IFN-I composite score provides a reproducible and scalable translational biomarker. By combining shared reference materials, calibrated thresholds, rigorous quality control, and transparent reporting, laboratories can achieve traceable and comparable measurements across centers, when implementing this six-gene qPCR-based IFN-I workflow, enabling broader multicenter studies and routine clinical deployment.

## Figures and Tables

**Table 1:** Baseline characteristics of the study population.

Characteristic	Value
Total participants, *n*	95
Sex, *n* (%)	Male: 53 (55.8%); Female: 42 (44.2%)
Age (years)	Median: 31.32 • Range: 18 days–89 years
Age categories, *n* (%)	0–2 years: 5 (5.3) • 3–10 years: 18 (18.9) • 11–18 years: 19 (20) • 19–40 years: 19 (20) • 41–65 years: 28 (29.5) • >65 years: 6 (6.3)
Encounter type, *n* (%)	Outpatient: 75 (78.9); Inpatient: 20 (21.1)
Main reason for evaluation	Routine blood tests; pre-surgical assessments; pediatric growth/metabolic monitoring; general health checks
Comorbidity status	No inflammatory, autoimmune, oncologic, metabolic, or endocrine diseases; minor non-inflammatory conditions only if present
Medication status	No immunomodulatory, corticosteroid, or hormonal therapy
Recent infection or vaccination (past 4 weeks)	None documented; no acute infections; no recent COVID-19 positivity

Age categories were defined a priori to reflect pediatric, young adult, middle age, and older adult strata relevant to IFN-I biology. *n:* number.

## Data Availability

The results of this article will be shared at the aggregate/population level upon reasonable request to the corresponding author.

## Supplementary Materials

The following supporting information can be downloaded at: https://www.mdpi.com/article/10.3390/medicina61122230/s1. Supplementary Table S1: Multivariable linear regression analysis of log-transformed ISG expression values (IFI27, IFI44L, IFIT1, ISG15, RSAD2, SIGLEC1) and of the composite IFN-I score.
