# Supplementary material for: Age- and Sex-Dependent Variation in the Type I Interferon Signature of Healthy Individuals
Source: Medicina (Kaunas). 2025 Dec 17;61(12):2230. doi: 10.3390/medicina61122230 (PMC12735248; doi:10.3390/medicina61122230)
Supplement: Supplementary file 1 [file medicina-61-02230-s001.zip › medicina-4034118-supplementary.pdf]

Supplementary Table S1: *Multivariable linear regression analysis of log-transformed ISG expression values (IFI27, IFI44L, IFIT1, ISG15, RSAD2, SIGLEC1) and of the composite IFN-I score.*

Gene (log-transformed)	β_age	p_age	β_sex (M–F)	p_sex	R^2
**IFIT1**	−0.000918	0.3682	−0.04014	0.4052	0.017
**IFI44L**	−0.00458	**0.0006**	−0.1303	**0.0346**	0.167
**IFI27**	+0.005972	**0.0125**	−0.04025	0.7178	0.067
**ISG15**	+0.0003438	0.7575	−0.06945	0.1892	0.019
**RSAD2**	−0.00001576	0.9914	−0.07469	0.2812	0.013
**SIGLEC1**	−0.0003653	0.7780	−0.09938	0.1071	0.030
**IFN Score**	−0.0001034	0.9234	−0.06790	0.1842	0.020

Each model includes age (continuous) and sex (binary: 0 = female, 1 = male) as covariates. Regression coefficients, 95% confidence intervals, p-values, and model R^2 are reported. These analyses were performed as a sensitivity assessment to determine whether age–ISG associations persisted after adjusting for sex and to quantify any independent demographic effects. Only IFI27 and IFI44L retained significant age associations, and IFI44L showed a modest independent sex effect, whereas no demographic variables influenced the other ISGs or the IFN-I score.
